# 950. Impact of the COVID 19 pandemic on antimicrobial stewardship program, broad-spectrum antibiotics consumption and *Clostridioides difficile* diarrhea incidence

**DOI:** 10.1093/ofid/ofac492.793

**Published:** 2022-12-15

**Authors:** Jose Luis Lamas Ferreiro, Judith Álvarez Otero, Ana Sanjurjo Rivo, Ignacio Enríquez de Salamanca Holzinger, Jorge Cavero, Jose Carlos de Miguel Bouzas, María Fernández Soneira, Irene Rodríguez Conde, Emilia Fernández Fernández, Marta Costas Vila, Javier de la Fuente Aguado

**Affiliations:** Ribera POVISA Hospital, Vigo, Galicia, Spain; Hospital Povisa, Vigo, Galicia, Spain; Hospital Povisa, Vigo, Galicia, Spain; Hospital Povisa, Vigo, Galicia, Spain; Hospital Povisa, Vigo, Galicia, Spain; Hospital Povisa, Vigo, Galicia, Spain; Hospital Povisa, Vigo, Galicia, Spain; Hospital Povisa, Vigo, Galicia, Spain; Hospital Povisa, Vigo, Galicia, Spain; Hospital Povisa, Vigo, Galicia, Spain; Hospital Povisa, Vigo, Galicia, Spain

## Abstract

**Background:**

The aim was to evaluate the impact of the COVID 19 pandemic on the antimicrobial stewardship program of our hospital, analyze changes in broad-spectrum antibiotics consumption, and analyze the evolution of the incidence of *Clostridioides difficile* (CD) diarrhea.

**Methods:**

Database with the following variables was created: monthly percentage of broad-spectrum antibiotic prescriptions that were evaluated by the antimicrobial stewardship team (AMST), monthly consumption of antimicrobials and monthly incidence of diarrhea due to *CD*. Pre-pandemic period was considered from March 1th, 2018 to February 29th, 2020 and the pandemic period from March 1th, 2020 to February 28th, 2022.

Time series analysis was performed with ARIMA models to assess the association of the pandemic period with a change in the monthly activity of AMST, in the monthly antibiotic consumption, and in the monthly incidence of *CD* diarrhea.

The correlation of the percentage of monthly broad-spectrum antibiotic prescriptions reviewed by AMST with the monthly broad-spectrum antimicrobials consumption was also evaluated, using the Spearman coefficient.

**Results:**

During the pandemic period, there was a significant reduction in monthly percentage of broad-spectrum antibiotic prescriptions reviewed by the AMST (28% vs 82%; P< 0.01). There was a 29% increase in consumption of broad-spectrum antibiotics in the pandemic period (15.7 vs 12.1 DDD per 100 bed-days; P=ns). The following antibiotics showed a significant increase in their consumption: antipseudomonal carbapenems (2 vs 1.4 DDD per bd; P< 0.01), daptomycin (1.8 vs 0.5 DDD per bd; P< 0.01), cefepime (1.1 vs 0.6 DDD per bd; P< 0.01), aztreonam (0.4 vs 0.3 DDD per bd; P=0.04), antibiotics with anti-MRSA activity (35.6 vs 12.9 DDD per bd; P< 0.01). There was a 41% increase in the incidence of nosocomial CD diarrhea (1.02 vs. 0.7 cases per 1000 bd; P=0.03). The percentage of broad-spectrum antibiotic prescriptions reviewed by the AMST correlated well with the consumption of this group of antibiotics (cc -0.63; P< 0.01).

**Conclusion:**

COVID-19 pandemic has had a significant impact on the antimicrobial stewardship program at our hospital, with an increase in broad-spectrum antimicrobials consumption and a significant increase in the incidence of *Clostridioides difficile* diarrhea.

**Disclosures:**

**All Authors**: No reported disclosures.

